# Cancer-specific variation in emergency presentation by sex, age and deprivation across 27 common and rarer cancers

**DOI:** 10.1038/bjc.2015.52

**Published:** 2015-03-03

**Authors:** G A Abel, J Shelton, S Johnson, L Elliss-Brookes, G Lyratzopoulos

**Affiliations:** 1 Cambridge Centre for Health Services Research, Department of Public Health and Primary Care, Institute of Public Health, Forvie Site, Robinson Way, Cambridge CB2 0SR, UK; 2 Care Quality Commission, Finsbury Tower, 103–105 Bunhill Row, London EC1Y 8TG, UK; 3 National Cancer Intelligence Network (NCIN), Public Health England, 5th Floor, Wellington House, 135-155 Waterloo Road, London SE1 8UG, UK; 4 Health Behaviour Research Centre, Department of Epidemiology and Public Health, University College London, 1-19 Torrington Place, London WC1E 6BT, UK

**Keywords:** emergency, diagnosis, route, age, sex, deprivation, inequalities

## Abstract

**Background:**

Although overall sociodemographic and cancer site variation in the risk of cancer diagnosis through emergency presentation has been previously described, relatively little is known about how this risk may vary differentially by sex, age and deprivation group between patients with a given cancer.

**Methods:**

Data from the *Routes to Diagnosis* project on 749 645 patients (2006–2010) with any of 27 cancers that can occur in either sex were analysed. Crude proportions and crude and adjusted odds ratios were calculated for emergency presentation, and interactions between sex, age and deprivation with cancer were examined.

**Results:**

The overall proportion of patients diagnosed through emergency presentation varied greatly by cancer. Compared with men, women were at greater risk for emergency presentation for bladder, brain, rectal, liver, stomach, colon and lung cancer (e.g., bladder cancer-specific odds ratio for women vs men, 1.50; 95% CI 1.39–1.60), whereas the opposite was true for oral/oropharyngeal cancer, lymphomas and melanoma (e.g., oropharyngeal cancer-specific odds ratio for women vs men, 0.49; 95% CI 0.32–0.73). Similarly, younger patients were at higher risk for emergency presentation for acute leukaemia, colon, stomach and oesophageal cancer (e.g., colon cancer-specific odds ratio in 35–44- *vs* 65–74-year-olds, 2.01; 95% CI 1.76–2.30) and older patients for laryngeal, melanoma, thyroid, oral and Hodgkin's lymphoma (e.g., melanoma specific odds ratio in 35–44- *vs* 65–74-year-olds, 0.20; 95% CI 0.12–0.33). Inequalities in the risk of emergency presentation by deprivation group were greatest for oral/oropharyngeal, anal, laryngeal and small intestine cancers.

**Conclusions:**

Among patients with the same cancer, the risk for emergency presentation varies notably by sex, age and deprivation group. The findings suggest that, beyond tumour biology, diagnosis through an emergency route may be associated both with psychosocial processes, which can delay seeking of medical help, and with difficulties in suspecting the diagnosis of cancer after presentation.

A substantial proportion of cancer patients are diagnosed through an emergency route (hereafter described as emergency presentation) (Elliss-Brookes *et al*, 2012; Gunnarsson *et al*, 2013). Reducing this proportion is desirable for at least three reasons. First, diagnosis of cancer through an emergency presentation is associated with poorer survival outcomes (Sjo *et al*, 2009; Elliss-Brookes *et al*, 2012; McPhail *et al*, 2013). Second, although being diagnosed with cancer is typically a stressful life event in itself, diagnosis through an emergency presentation increases stress, anxiety and inconvenience for patients and their relatives. Third, because patients diagnosed through emergency presentations typically require urgent management, pressure on the out-of-hours capacity of the health-care system is intensified, and may itself contribute to poorer outcomes (Iversen *et al*, 2008; McPhail *et al*, 2013).

Despite a justifiable desire to reduce emergency presentations, there is uncertainty about how this can be achieved. Some emergency presentations may reflect purely biological factors: tumour type, aggressiveness or anatomical location may lead to sudden and critical clinical presentations with minimal or no prodromal symptoms (and, therefore, no opportunity for prior contact with the formal health-care system). In such circumstances, emergency presentations can be deemed unavoidable (Lyratzopoulos *et al*, 2014). However, in other patients, symptoms would have preceded the emergency presentation, but patients themselves or their doctors may not have promptly appreciated their likely importance (Lyratzopoulos *et al*, 2014). Therefore, diagnoses through emergency presentation may, in addition to tumour biology, reflect psychosocial factors preventing prompt presentation and help-seeking (Robb *et al*, 2009; Beeken *et al*, 2011), or diagnostic difficulty by doctors in the presence of atypical symptoms (Hamilton, 2012; Sheringham *et al*, 2014).

Although the exact contribution of biological, patient and health-care factors is unknown, studies have documented that many patients diagnosed with cancer through an emergency presentation have had prior general practitioner consultations (Barrett and Hamilton, 2008), often for atypical symptoms (Cleary *et al*, 2007; Sheringham *et al*, 2014), but some have had no prior contact with primary care. Therefore, potential reductions in the frequency of emergency presentations may be achievable both by improving the effectiveness of the diagnostic process during primary care encounters and by reducing the proportion of patients who are diagnosed as emergencies without prior help-seeking.

Insights into potential responsible mechanisms may be obtained by appreciating variation in the risk of emergency presentation. Large variations in this risk by cancer diagnosis, age and socioeconomic status have been previously reported (Elliss-Brookes *et al*, 2012; National Cancer Intelligence Network, 2014). However, for patients with a given cancer, the risk of emergency presentation may differ by patient characteristic, and variably so for different cancers. Appreciating such variation may further help identify the mechanisms contributing to these risks, which can then be targeted by interventions. Against this background we aimed to examine cancer-specific variation in emergency presentation by sex, deprivation group and age among patients with common and rarer cancers that can occur in either sex.

## Materials and methods

### Data

Data were extracted from the National Cancer Data repository for England. The methods used to assign the route to diagnosis have been described in detail previously (Elliss-Brookes *et al*, 2012; National Cancer Intelligence Network, 2014). Briefly, all patients diagnosed with cancer during the study period (2006–2010) were assigned to a diagnostic ‘route'. ‘Routes' can be conceptualised as different pathways to diagnosis. They included emergency presentation and other routes, such as primary care referral for suspected cancer (either a ‘2-week wait' or routine referral) and screening.

Using cancer registration records as the base data set, additional information from Hospital Episodes Statistics, National Cancer Waiting Times and National Health Service screening programmes data (for breast, bowel and cervical cancers) were linked at person-tumour level. The emergency presentation route denotes a new diagnosis of cancer after an emergency hospital admission or Accident and Emergency department attendance, or after emergency hospital transfer or emergency general practitioner referral; detailed operational definitions and algorithms have been further stipulated in detail (Elliss-Brookes *et al*, 2012; National Cancer Intelligence Network, 2014).

For the present study, data relate to patients aged 25 years or older with one of 27 non-sex-specific malignancies, aiming for the largest possible number of cancers that can occur in either sex with an adequate sample size – that is, anal, bladder, brain, colon, Hodgkin's lymphoma, laryngeal, liver, lung, melanoma, mesothelioma, multiple myeloma, non-Hodgkin's lymphoma, thyroid, oesophageal, oral, oropharyngeal, renal, rectal, pancreatic, small intestine, soft-tissue sarcoma, stomach and cancer of unknown primary, in addition to four leukaemia groups (acute lymphoblastic leukaemia (ALL), acute myeloid leukaemia (AML), chronic lymphocytic leukaemia (CLL) and chronic myeloid leukaemia (CML)). Diagnostic code definitions as per the International Classification of Diseases-10 for each diagnostic group are provided in Supplementary Online Material 1. Information was also available for age at diagnosis, sex, year of diagnosis and area-based deprivation of patients' home address (Index of Multiple Deprivation 2010, income domain only, Department for Communities and Local Government, 2011). Ages were aggregated into 10-year age groups, apart from those over 85 years, which were treated as a single group (after excluding those aged over 99 following the study by Elliss-Brookes *et al*, 2012). Deprivation was split into five groups on the basis of national quintiles.

### Analysis

We first described the crude proportion of patients who were diagnosed through emergency presentation by variable category (i.e., by age group, sex, deprivation group, year of diagnosis and cancer diagnosis) and calculated the respective crude odds ratios. Adjusted odds ratios were estimated using a logistic regression model that, conditional on being a cancer case, predicts the emergency presentation status (yes/no binary variable) adjusting for age, sex, deprivation group, year of diagnosis and cancer (model 1).

Subsequently, we examined whether the effect of sex, deprivation and age varied between cancers by including two-way interactions between cancer and each of the three sociodemographic characteristics in the above model. Each of the three interaction terms was tested in turn with a single joint test. Statistically significant interaction terms (*P*<0.05) were retained in the model (model 2), which was then used to estimate cancer-specific odds ratios for emergency presentation, by sex, age and deprivation. When all three interaction terms are retained, this model is almost equivalent to a series of models stratified by cancer (except for the shared effect of year across cancers). This model was also used to predict the risk of emergency presentation for all-cancer–age–sex–deprivation group strata for cases diagnosed in 2010.

In both multivariable models the presence of overdispersion was assessed by the Pearson *χ*^2^-statistic and by examination of the distribution of deviance residuals. Overdispersion was then accounted for by scaling the standard errors appropriately. All analyses were carried out in Stata v13.1 (StataCorp, College Station, TX, USA).

## Results

There were 749 645 incident tumours contained within the 27 non-sex-specific cancer diagnosis groups under consideration; among those the diagnosis was through an emergency presentation in 232 281 (31%) cases (Table 1). This percentage is higher than previously published ‘all-cancer' averages (Elliss-Brookes *et al*, 2012; National Cancer Intelligence Network, 2014) primarily because of the exclusion of the sex-specific sites (including prostate and breast cancers, two common cancers with low proportions of emergency presentations).

### Overall variation in emergency presentation by age, sex, deprivation, diagnosis year and cancer (model 1 findings)

There was strong evidence for variation in emergency presentation by sex, deprivation group, age, year of diagnosis and cancer diagnosis (*P*<0.0001 for all; Table 1). Specifically, there was a small excess risk of emergency presentation among women compared with men (adjusted odds ratio, 1.06; 95% CI 1.04–1.08). The risk of emergency presentation increased with each increasing deprivation group (adjusted odds ratio for most *vs* least deprived group, 1.55; 95% CI 1.51–1.59). Crudely, the risk of emergency diagnosis increased with age across all ages considered. However, after adjusting for other variables there was a slight increase in risk with decreasing age for those under 55 years, but those most at risk were still the older patients (e.g., adjusted odds ratio for ages 75–84 *vs* 65–74 years, 1.47; 95% CI 1.45–1.50).

The crude risk of emergency presentation decreased year-on-year, with diagnosis year being highest (33%) at the baseline year (2006) and lowest (30%) in the last year (2010) of the study period (*P*<0.0001). A consistent pattern was seen in the adjusted findings.

There was very large variation in the crude risk for emergency presentation by cancer, being lowest for melanoma and oral cancer (2% and 5%, respectively) and very high for patients with brain cancer and ALL (67% and 79%, respectively; Table 1). This large variation persisted after adjustment for other variables, with about a 180-fold variation in the adjusted odds of emergency presentation across cancer diagnoses (i.e., odds ratio comparing ALL to melanoma 7.19/0.04=179.31).

### Cancer-specific variation in emergency presentation by age, sex and deprivation (model 2 findings)

**Figure 1:**
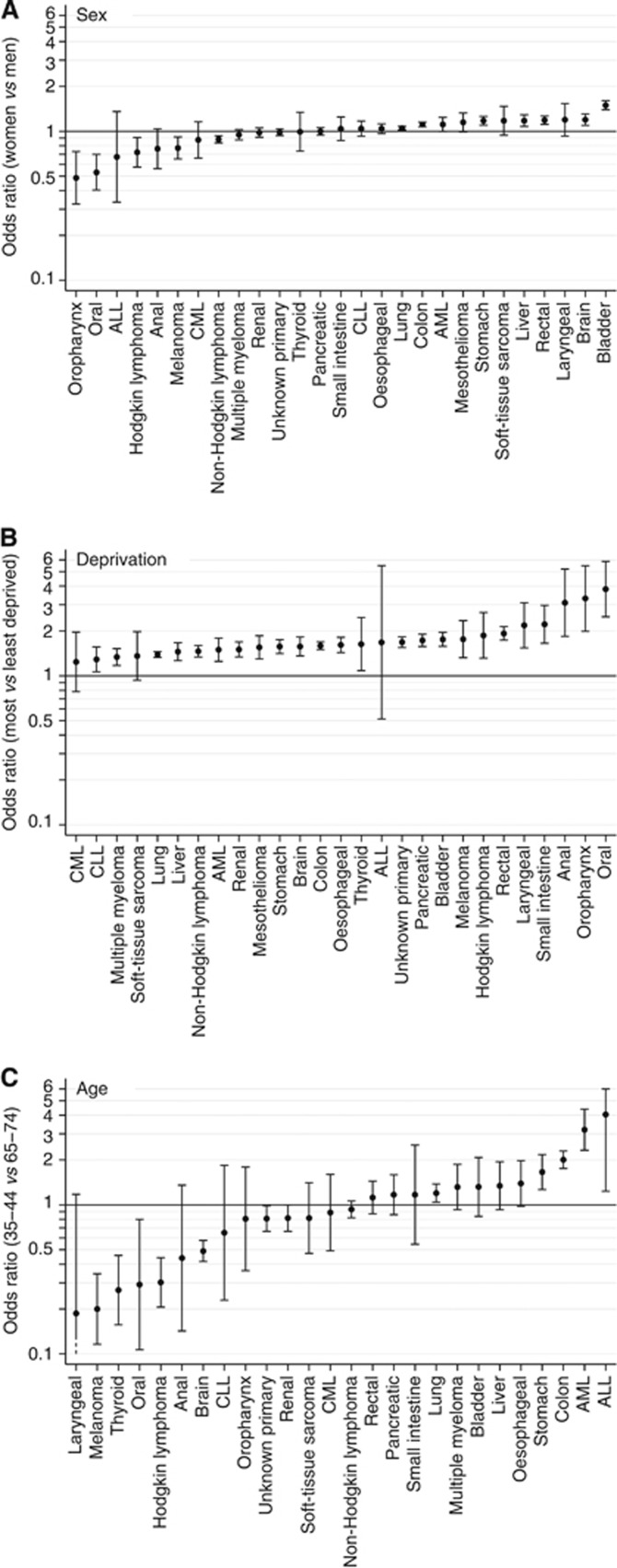
**Cancer-specific odds ratios and 95% confidence intervals for emergency presentation comparing (**A**) women *vs* men; (**B**) most *vs* least deprived patients; and (**C**) 35–44-year-olds *vs* 65–74-year-olds.** Note that mesothelioma is not displayed in **C**, see Figure 2 for details.

There was very strong evidence (*P*<0.0001 for each of the three two-way interaction terms) that the effect of sex, age and deprivation varied between the studied cancers. Figure 1 shows cancer-specific sex, age (35–44 *vs* 65–74 years) and deprivation (most *vs* least deprived) associations. Note that, although a modest amount of overdispersion was present (resulting in an increase in the width of confidence intervals of 31%), the distribution of deviance residuals was very close to normal. Variability between cancers is clearly largest for the effect of age; furthermore, the largest age effects tend to be larger than either the sex or deprivation effects. We discuss each of the sociodemographic variables in detail below.

#### Sex

There was evidence (*P*<0.05) that women had greater risk of emergency presentation than men for seven cancers – namely, in descending order of effect size, bladder, brain, rectal, liver, stomach, colon and lung cancer (odds ratios for women *vs* men ranging from 1.50 to 1.05 for bladder and lung cancer, respectively; Figure 1A). Conversely, there was evidence (*P*<0.05) that women had lower risk of emergency presentation compared with men for five cancers – namely, in descending order of effect size, oropharyngeal, oral, Hodgkin's lymphoma, melanoma and non-Hodgkin's lymphoma (odds ratios for women *vs* men ranging from 0.49 to 0.88 for oropharyngeal and non-Hodgkin's lymphoma, respectively). There was no evidence for variation by sex in the risk of emergency presentation for the other 12 cancers examined; for some of those cancers this may reflect the small number of patients concerned.

#### Deprivation

There was evidence (*P*<0.05) that patients in the most deprived groups were at greater risk of emergency presentation in nearly all (24 out of 27) cancers (Figure 1B). Within this overall consistent pattern, excess risk of emergency presentation among more deprived patients was particularly pronounced for patients with five cancers – namely, oral, oropharyngeal, anal, small intestine and laryngeal cancers (odds ratios (most *vs* least deprived patients) ranging from 3.82 to 2.18 for patients with oral and laryngeal cancer, respectively). The three cancers without evidence for variation by deprivation were ALL, CML and soft-tissue sarcoma – the first of which had very few cases and hence very large uncertainty around the estimate.

#### Age

**Figure 2:**
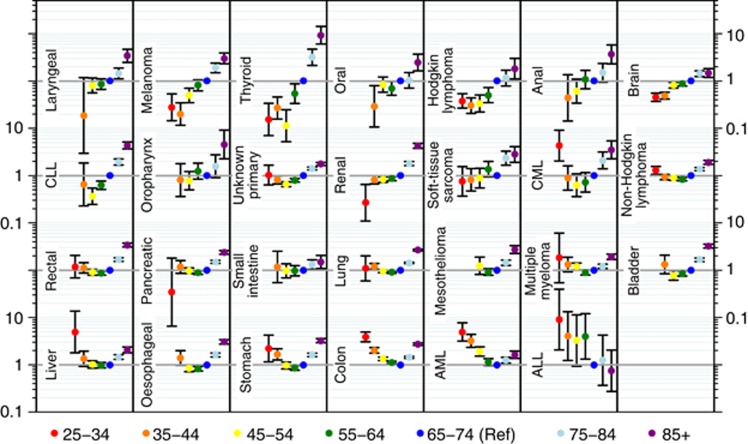
**Cancer-specific odds ratios and 95% confidence intervals for emergency presentation by age group compared with 65–74 years (reference).** Note that where cancer-specific age groups contained no cases or all cases were either emergency or non-emergency presentations, odds ratios cannot be estimated and are not shown. This relates to the two younger age groups (25–34 or 35–44), for a total of 30 individual tumours across nine cancers.

Although on average older patients had higher risk of emergency presentation, the presence, size and direction of associations with age varied notably between patients with different cancers. The comparison of 35–44-year-olds with 65–74-year-olds is useful as a direct comparison with sex and deprivation effects (Figure 1C), but for a full understanding of the cancer-specific age patterns variation across all age groups ought to be considered (Figure 2). Three patterns can be initially distinguished. For some cancers, the risk of emergency presentation increases with age across all age groups (laryngeal, melanoma, thyroid, oral, anal, brain, oropharynx, renal cancer and soft-tissue sarcoma). For one cancer (ALL), the reverse pattern was observed. For the remaining cancers (AML, colon, stomach, oesophageal, liver, bladder, multiple myeloma, mesothelioma, lung cancer, small intestine, pancreatic, rectal, non-Hodgkin's lymphoma, CLL, CML and cancer of unknown primary), either a J- or a U-shaped relationship with age was apparent, such that the risk of emergency presentation initially decreased and then increased with age, with the oldest patients in most cases being those most at risk. There was wide variability in the overall size of age effects, which were particularly large for cancers such as thyroid, melanoma and ALL, decreasing to almost no age effect for small intestine cancer.

**Figure 3:**
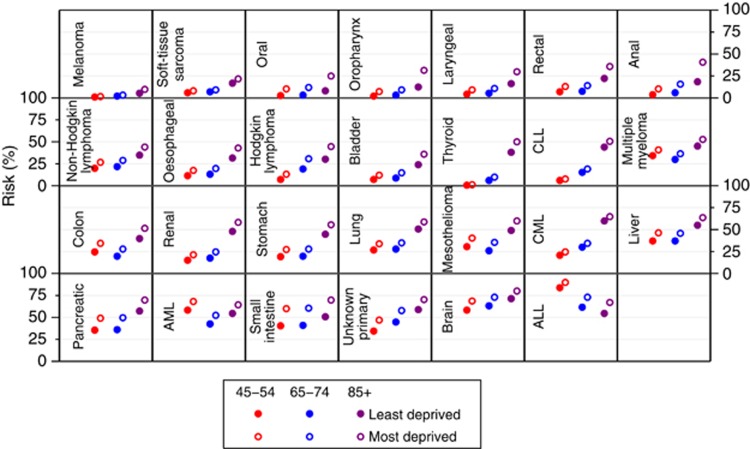
**Predicted risk of diagnosis of cancer through an emergency presentation for men in three age groups (45–54, 65–74 and 85+ years), two deprivation groups (most and least deprived) and with 27 cancers.** Confidence intervals can be found in Supplementary Online Material 2.

The appreciable size of cancer-specific sociodemographic variation in the risk of emergency presentation is further illustrated by the predicted risk of emergency presentations. This is shown in full for each cancer–age–sex–deprivation group strata for cases diagnosed in 2010 in Supplementary Online Material 2. We also show the predicted risk for men for three age groups (45–54, 65–74 and 85–99) and two deprivation groups (least and most deprived; Figure 3). In particular, we note that the sociodemographic variation introduces considerable within-cancer variation. For example, for thyroid, renal, pancreatic and ALL, we see that the risk of diagnosis through an emergency presentation between extreme the age group-deprivation strata shown in Figure 3 varies between 1% and 50%, 15% and 58%, 36% and 70% and 55% and 90%, respectively.

## Discussion

We describe notable cancer-specific differences in the risk of emergency presentation by patient characteristic among patients with 27 common and rarer cancers that can occur in either sex. Despite the study period being relatively short (i.e. 5 years), we also observed a decreasing trend in emergency presentations over time.

### Comparisons with previous evidence (what is known about the subject and what does this study add)

Although previous work has addressed variation in the risk of emergency presentation by cancer site and sociodemographic characteristics, our study examines how this risk varies for patients with a given cancer but with different sex, age and deprivation status.

Regarding overall patterns of variation in the risk of emergency presentation by cancer, with few differences in diagnostic group definitions, the findings iterate previous evidence encompassing between 15 and 56 cancers (Elliss-Brookes *et al*, 2012; National Cancer Intelligence Network, 2014). However, in this study we have demonstrated that variation by cancer persists after adjusting for age, sex and deprivation. Relatedly, it should be noted that ‘linear' crude patterns of decreasing risk for emergency presentation in younger age groups disappear after case-mix adjustment (Table 1). This is chiefly a demonstration of the differential age case-mix of various cancer sites with, for example, melanoma (the site with the lowest proportion of emergency presentations) being relatively common in younger age groups (28% of all 25–44-year-olds with non-sex-specific cancers included in this study). Similarly, the higher odds of emergency presentation in women were substantially attenuated after case-mix adjustment; in contrast, there was little apparent confounding of deprivation differences in risk of emergency presentation by other variables (Table 1). The present study, additionally, describes time trends (year-on-year) more clearly and considers colon and rectal cancer separately for the first time, indicating that the proportion of emergency presentation is two-fold greater for patients with colon compared with rectal cancer. Symptom signatures of rectal and colon cancer are likely to vary – as also shown for other colorectal cancer subsites (Hamilton, 2005). Another study also indicates that colon cancer is associated with substantially higher proportion of multiple pre-referral consultations compared with rectal cancer after presentation in primary care (Lyratzopoulos *et al*, 2012).

Regarding sociodemographic predictors of emergency presentation, the findings reiterate previously reported patterns of variation both from the *Routes to Diagnosis* project and from other settings, indicating higher risk of emergency presentation among older patients, those with lower socioeconomic status, and among women (Rabeneck *et al*, 2006; Bottle *et al*, 2012; Elliss-Brookes *et al*, 2012; National Cancer Intelligence Network, 2014; Wallace *et al*, 2014). However, this study also demonstrates that the presence, size and direction of sociodemographic differences in emergency presentation vary considerably between patients with a given cancer.

### Strengths and limitations

Our study uses population-based data of high quality for many different cancers and a relatively recent study period, and validated methodologies to define and assign the diagnosis of cancer through an emergency presentation. Sample attrition due to missing sociodemographic or cancer diagnosis information was trivial, given the very high completeness of this information in the primary sources used in the *Routes to Diagnosis* project. A small but non-trivial proportion of cases (4%) have been assigned an ‘unknown' route, as information was not available about the diagnosis route for these tumours from routine data sets. These have been treated as ‘non-emergency presentations' for the purposes of this analysis, as with our operational definition it is impossible to have an emergency presentation without having a signal in Hospital Episode Statistics. There was no information on symptom at presentation, nor about the exact circumstances preceding emergency presentation – more specifically, whether the presentation was the first ever contact with the health-care system, or whether patients had previously consulted for symptoms either in primary or in secondary care.

### Interpretation and implications

The *Routes to Diagnosis* project reveals a wealth of evidence that allows for a highly refined and stratified understanding of the risk of emergency presentation, not only by sociodemographic characteristics and cancer but also by their interactions. Given the large size and multiple types of variation revealed, it would be imprudent to attempt to ‘explain' all observed findings where notable variation was detected. Instead we propose that appreciation of the large and complex nature of the variations we describe should motivate future clinical, epidemiological and behavioural science research. We nonetheless provide a few hypotheses about some aspects of the findings. Two observations can help to inform the consideration of the findings.

First, most known sociodemographic inequalities in markers of diagnostic timeliness (such as stage at diagnosis) are concentrated on cancers with relatively clear-cut symptom signatures—that is cancers in which the majority of patients present with visible, palpable or audible symptoms (Lyratzopoulos and Abel, 2013; Lyratzopoulos *et al*, 2013a; Robbins *et al*, 2014). These include melanoma, oral/oropharyngeal, anal and laryngeal cancers (and also endometrial, breast and testicular cancer, not examined here as sex-specific cancers). Bearing this observation in mind it is apparent that, where present, cancer-specific inequalities (by sex, deprivation and age) tend to relate to cancers characterised by clear-cut symptoms in most patients (i.e., oral/oropharygeal cancer – oral ulceration/lesion; melanoma – visible skin lesion; anal – anal ulceration/lesions; and laryngeal – voice hoarseness). For example, cancer-specific variation in risk of emergency presentation by deprivation group was particularly pronounced for oral, oropharyngeal and laryngeal cancers. These patterns would seem to indicate a reduced ability or motivation by poorer patients to appropriately appraise and seek medical help (Walter *et al*, 2012) for easily noticeable bodily changes compared with more affluent patients, in turn prolonging the patient interval and increasing the risk of emergency presentation (Lyratzopoulos *et al*, 2014). Variation in the risk of emergency presentation by deprivation was, among others, pronounced for anal and rectal cancer, which might suggest a degree of socioeconomic patterning of stigma or embarrassment associated with gastrointestinal symptoms.

Second, although emergency presentations are multifactorial, they do seem to represent a marker of ‘diagnostic difficulty' after presentation. This becomes apparent when appreciating that ‘harder-to-suspect' cancers (i.e., those associated with the highest proportions of multiple pre-referral primary care consultations, such as multiple myeloma, stomach, pancreatic and colon cancer) also have a high proportion of emergency presentations (Table 1) (Hamilton, 2012; Lyratzopoulos *et al*, 2012, 2013b). Conversely, ‘easier-to-suspect' cancers (such as melanoma and breast cancer) have the lowest proportion of emergency presentations (Elliss-Brookes *et al*, 2012; National Cancer Intelligence Network, 2014). Taking these observations into account, it can be noted that bladder cancer is associated with notable sex differences in the promptness of diagnosis after presentation, betraying a higher level of difficulty by doctors in suspecting the diagnosis of bladder cancer in symptomatic women (Lyratzopoulos *et al*, 2013c), and it is for bladder cancer that the highest excess risk of emergency presentation for women was also observed. It is therefore plausible that the excess risk of emergency diagnosis of women with bladder cancer reflects prolonged intervals to diagnosis after presentation.

Finally, there is a group of cancers for which men are at greater risk of diagnosis through an emergency presentation – for example, oral and oropharyngeal cancer (both characterised by clear-cut symptoms (oral ulceration/lesion) in most patients) and melanoma (visible skin lesions in most patients)). These observations would seem to suggest reduced levels of body consciousness and reduced ability to appraise and seek help for easily recognisable symptoms among men compared with women.

As remarked in the Results section, cancer-specific variation in risk of emergency presentation by age was much larger compared with variation by sex and deprivation. We would posit that, given its very large size, the greatest part of this variation reflects biological factors (i.e., differences in tumour type and aggressiveness between patients with cancers of the same site but in different age groups). However (bearing in mind that the excess risk in older patients was non-uniform between cancers but present in all but one; Figure 2), reduced help-seeking in older age may be another likely source, given lower levels of awareness of age-related risk of cancer and reduced knowledge about likely cancer symptoms in older individuals (Robb *et al*, 2009; Forbes *et al*, 2013). In addition, comorbidity (including mental health comorbidity, e.g., dementia syndromes) and/or social isolation/lack of support in older age may be contributing to excess risk of diagnosis through an emergency presentation – for the cancers in which such patterns are observed (Wallace *et al*, 2014). Conversely, younger patients had excess risk of emergency presentation for some cancers (e.g., CML, liver, colon, AML and ALL). Such instances may betray the greater difficulty in suspecting the diagnosis of these cancers in younger patients post presentation to a general practitioner (Lyratzopoulos *et al*, 2012), although why this pattern is apparent only for some cancers (Figure 2) should be the subject of further investigation.

We would like to emphasise that epidemiological data such as used in this study cannot determine the potential for avoiding the events of interest (emergency presentations), nor can they help accurately ‘partition' the contribution of tumour, patient and health-care factors in emergency presentation. Moreover, this study cannot determine which, or indeed what, proportion of emergency diagnosis may have been avoided, and how; further epidemiological research and retrospective case reviews are needed in this area (Lyratzopoulos *et al*, 2014). However, were all emergency diagnoses a manifestation of tumour factors, we would not be expecting to see the degree of cancer-specific variations by the sociodemographic variables that we have observed. Interventions should aim to reduce the proportion of emergency presentations by reducing the proportion that can be attributable to either health-care or patient factors.

The patterns of variation that we observe provide aetiological insights into factors other than tumour biology implicated in emergency presentations. They can therefore help to motivate and target the development and evaluation of interventions with a public health or health-care focus. We provide the research and policy-making community with ample evidence about the variable (stratified) risk of cancer diagnosis through an emergency presentation, which can be used to inform targeted interventions for different cancers/sociodemographic groups. As an example, among patients with oral or oropharyngeal cancer the risk of emergency presentation is higher for men, further increasing notably with increasing deprivation (Figure 1A and B). Future early diagnosis campaigns for cancers of the oral cavity can therefore focus on men from deprived communities. Cancers of the oral cavity are also known to be associated with the longest median patient interval compared to another 17 cancers (Keeble *et al*, 2014).

We also wish to note the particularly high proportion of patients with colon cancer who are diagnosed as emergencies (31%)—two-fold greater than for rectal cancer. An effective cancer control strategy for this common cancer is increasing the uptake of screening, which can be expected to decrease emergency presentations from colon cancer (Pruitt *et al*, 2014).

In conclusion, we have provided a comprehensive account of the descriptive epidemiology of cancer diagnosis through emergency presentation for patients with 27 different cancers, examining both overall and cancer-specific variation by patient characteristic. The findings suggest that, beyond tumour biology, emergency diagnosis may be associated with psychosocial processes delaying presentation and diagnostic difficulty post presentation; our findings also ‘map out' the potential variable influence of patient (psychosocial), health-care (diagnostic difficulty) and tumour-related factors. They can help motivate further research priorities and policy initiatives targeted at patient groups at greater risk of emergency presentation. Notable improvements over a short period of time have also been observed, indicating that to an extent the potential for decreasing emergency presentations is already being harvested.

## Figures and Tables

**Table 1:** Crude proportion, and crude and adjusted odds ratios for diagnosis through emergency presentation, by age group, sex, deprivation group, year of diagnosis and cancer diagnosis

		**Emergency Presentation**		**Odds ratio (95% CI)**^a^	
	**All cases**	**Number**	**%**	**Crude**	**Adjusted**
All Cases	749 645	232 281	31.0%	N/A	N/A
**Age**					
25–34	8586	1659	19.3%	0.65 (0.62–0.69)	1.10 (1.00–1.21)
35–44	22 356	4481	20.0%	0.68 (0.66–0.70)	1.01 (0.95–1.07)
45–54	57 128	12 920	22.6%	0.79 (0.78–0.81)	0.94 (0.90–0.97)
55–64	140 074	33 741	24.1%	0.86 (0.85–0.87)	0.91 (0.89–0.93)
65–74	208 095	56 025	26.9%	Reference	Reference
75–84	218 951	77 723	35.5%	1.49 (1.47–1.51)	1.47 (1.45–1.50)
85+	94 455	45 732	48.4%	2.55 (2.51–2.59)	2.62 (2.56–2.69)
**Sex**					
Men	428 155	126 300	29.5%	Reference	Reference
Women	321 490	105 981	33.0%	1.18 (1.16–1.19)	1.06 (1.04–1.08)
**Deprivation group**					
1—Least deprived	138 269	36 255	26.2%	Reference	Reference
2	155 743	44 332	28.5%	1.12 (1.10–1.14)	1.09 (1.06–1.11)
3	157 940	48 256	30.6%	1.24 (1.22–1.26)	1.19 (1.16–1.22)
4	152 953	50 779	33.2%	1.40 (1.38–1.42)	1.33 (1.30–1.36)
5—Most deprived	144 740	52 659	36.4%	1.61 (1.58–1.64)	1.55 (1.51–1.59)
**Year of diagnosis**					
2006	146 259	47 987	32.8%	Reference	Reference
2007	147 473	46 333	31.4%	0.94 (0.92–0.95)	0.93 (0.91–0.96)
2008	152 125	46 784	30.8%	0.91 (0.90–0.92)	0.91 (0.89–0.93)
2009	153 600	46 890	30.5%	0.90 (0.89–0.91)	0.90 (0.88–0.92)
2010	150 188	44 287	29.5%	0.86 (0.84–0.87)	0.87 (0.85–0.89)
**Cancer diagnosis**					
Melanoma^b^	45 561	967	2.1%	0.03 (0.03–0.04)	0.04 (0.04–0.04)
Oral	9801	491	5.0%	0.08 (0.08–0.09)	0.09 (0.08–0.10)
Thyroid	8254	460	5.6%	0.09 (0.09–0.10)	0.11 (0.10–0.13)
Oropharynx	6429	365	5.7%	0.10 (0.09–0.11)	0.12 (0.10–0.14)
Anal	3381	345	10.2%	0.18 (0.16–0.20)	0.19 (0.16–0.22)
Laryngeal	8283	833	10.1%	0.18 (0.17–0.19)	0.20 (0.18–0.22)
Soft tissue sarcoma	4839	635	13.1%	0.24 (0.22–0.26)	0.26 (0.23–0.29)
Rectal	54 076	8177	15.1%	0.29 (0.28–0.29)	0.30 (0.28–0.31)
Hodgkin lymphoma	4768	674	14.1%	0.26 (0.24–0.29)	0.31 (0.28–0.35)
Bladder^b^	42 234	7834	18.5%	0.36 (0.36–0.37)	0.34 (0.33–0.35)
Oesophageal^b^	32 470	7062	21.7%	0.44 (0.43–0.46)	0.45 (0.43–0.47)
CLL	11 892	2950	24.8%	0.53 (0.51–0.55)	0.53 (0.49–0.56)
Renal^b^	29 469	7733	26.2%	0.57 (0.55–0.59)	0.62 (0.60–0.65)
Non-Hodgkin^b^ lymphoma	46 329	12 393	26.7%	0.58 (0.57–0.60)	0.64 (0.62–0.66)
Colon	97 880	30 777	31.4%	0.73 (0.72–0.75)	0.73 (0.71–0.75)
Stomach^b^	29 893	9913	33.2%	0.79 (0.77–0.82)	0.75 (0.73–0.78)
Multiple myeloma^b^	18 272	6693	36.6%	0.93 (0.90–0.96)	0.96 (0.91–1.00)
Mesothelioma	10 116	3631	35.9%	0.90 (0.86–0.93)	0.96 (0.90–1.02)
Lung^b^	162 543	62 498	38.5%	Reference	Reference
CML	1702	656	38.5%	1.00 (0.91–1.11)	1.01 (0.88–1.17)
Pancreatic^b^	33 295	16 364	49.1%	1.55 (1.51–1.58)	1.56 (1.51–1.62)
Liver	14 732	7270	49.3%	1.56 (1.51–1.61)	1.60 (1.52–1.68)
Unknown primary	43 290	24 805	57.3%	2.15 (2.10–2.19)	2.00 (1.94–2.07)
AML	9611	5388	56.1%	2.04 (1.96–2.13)	2.15 (2.02–2.29)
Small intestine	3399	1863	54.8%	1.94 (1.81–2.08)	2.15 (1.95–2.38)
Brain^b^	16 710	11 175	66.9%	3.23 (3.12–3.34)	3.96 (3.77–4.17)
ALL	416	329	79.1%	6.05 (4.78–7.67)	7.19 (5.08–10.16)

Abbreviations: ALL=acute lymphoblastic leukaemia; AML=acute myeloid leukaemia; CI=confidence interval; CLL=chronic lymphocytic leukaemia; CML=chronic myeloid leukaemia; NA=not applicable.

a *P*<0.0001 for all based on joint test of categorical variables. A modest amount of overdispersion was found resulting in an increase in the width of confidence intervals of 45%.

b See also Elliss-Brookes *et al*, 2012.
